# Visible-Light Photocatalytic Activity of a ZnO-Loaded Isoreticular Metal-Organic Framework

**DOI:** 10.3390/molecules30061375

**Published:** 2025-03-19

**Authors:** Ana Y. Rojas-Forero, Laura Y. Hernández-Benítez, María L. Ospina-Castro, Nataly J. Galán-Freyle, John R. Castro-Suarez, Maximiliano Méndez-López, Samuel P. Hernández-Rivera, José A. Centeno-Ortiz, Sandra P. Romero-Nieto, Leonardo C. Pacheco-Londoño

**Affiliations:** 1 Ingeniería Ambiental, Vicerrectoría de Investigación, Universidad ECCI, Bogotá 110231, Colombia; anrojasf@unal.edu.co (A.Y.R.-F.); laurayhb21@gmail.com (L.Y.H.-B.); 2 Grupo de Investigación Química Supramolecular Aplicada, Programa de Química, Universidad del Atlántico, Barranquilla 080001, Colombia; mariaospina@mail.uniatlantico.edu.co; 3 Facultad de Ciencias Básicas y Biomédicas, Centro de Investigación en Ciencias de la vida, Universidad Simón Bolívar, Barranquilla 080002, Colombia; nataly.galan@unisimon.edu.co; 4 Área Básicas Exactas, Universidad del Sinú, Seccional Cartagena, Cartagena 130015, Colombia; johnrcastro@unisinu.edu.co; 5 Grupo de Química y Biología, Departamento de Química y Biología, Universidad del Norte, Km 5 Vía Puerto Colombia, Barranquilla 080001, Colombia; maximilianom@uninorte.edu.co; 6 ALERT DHS Center of Excellence for Explosives Research, Department of Chemistry, University of Puerto Rico-Mayagüez Campus, Mayagüez, PR 00681, USA; samuel.hernandez3@upr.edu (S.P.H.-R.); jacenteno@comcast.net (J.A.C.-O.); 7 Ingeniería Mecánica, Vicerrectoría de Investigación, Universidad ECCI, Bogotá 111311, Colombia; sromeron@ecci.edu.co

**Keywords:** heterogeneous photocatalysis, metal-organic framework (MOF), semiconductor, nanotechnology, photodegradation, ZnO, organic pollutants

## Abstract

A hybrid material composed of IRMOF-3 and ZnO (IRMOF-3/ZnO) was synthesized to enhance photocatalytic methylene blue (MB) degradation under visible-light irradiation. Scanning electron microscopy, Fourier-transform infrared spectroscopy, X-ray diffraction, and diffuse-reflectance UV-Vis analyses confirmed the successful integration of ZnO into the IRMOF-3 framework. Compared with unmodified IRMOF-3, the hybrid demonstrated superior MB decomposition, as evidenced by faster reaction rate constants and shorter half-lives. Monitoring the MB absorbance at 670 nm (λ_max_) revealed more pronounced colorant removal when IRMOF-3/ZnO was exposed to a visible-light source. Diffuse-reflectance UV-Vis spectroscopy showed that IRMOF-3 has a band gap of 2.7 eV, whereas IRMOF-3/ZnO exhibits a slightly higher band gap of 2.8 eV. This modest shift, coupled with the strong interaction between the ZnO semiconductor and the MOF’s amine functionalities, enabled two distinct energy-transfer pathways: intermolecular transfer from IRMOF-3 linkers (acting as visible-light antennas) to ZnO, and intramolecular transfer from Zn to IRMOF-3. Together, these pathways generated abundant free radicals for efficient dye degradation. Despite the necessity for careful synthesis protocols and control of operating conditions to preserve the MOF structure and optimize ZnO loading, the IRMOF-3/ZnO hybrid shows promise as a robust, cost-effective photocatalyst for water-pollutant remediation, taking advantage of the more abundant visible region of solar light.

## 1. Introduction

Industrial wastewater discharge is a global environmental concern, as it degrades the quality and distribution of water resources, undermines ecosystem resilience, and jeopardizes human health, food security, and sanitation [1]. Textile and paper industries, in particular, produce effluents containing various dyes that may be toxic if ingested and can even exhibit carcinogenic properties. Because the natural self-purification mechanisms of water bodies cannot effectively remove these dye pollutants [2,3,4,5], light transmission in aquatic environments is obstructed, disrupting biochemical cycles and reducing dissolved oxygen availability. Consequently, water bodies develop poor organoleptic qualities and deteriorating landscapes [6]. Preventing wastewater discharge is, therefore, critical for safeguarding water resources against organic pollutants.

Traditional remediation techniques often struggle to degrade stable colorant molecules [2,7], necessitating more advanced approaches, such as advanced oxidation processes (AOPs). AOPs involve chemical or photochemical oxidation reactions [8,9,10,11,12,13,14,15,16,17,18] that generate free radicals—hydroxyl radicals, particularly—that degrade organic compounds [19,20]. Heterogeneous photocatalysis is a noteworthy example of AOPs, wherein a catalyst activated by a light source produces reactive radicals that mineralize organic dyes without generating secondary waste (e.g., sludge) [21]. Titanium dioxide (TiO_2_) has long served as the catalyst of choice for heterogeneous photocatalysis due to its UV-driven efficiency [21,22,23]. Nonetheless, numerous materials have been explored as alternative catalysts for dye degradation via photodegradation [24,25,26,27,28,29,30,31], including the use of solar (visible) light, which is safer and widely available, especially for low dye concentrations (≤1 ppm) [32].

Zinc oxide (ZnO) is among the most promising photocatalysts thanks to its low toxicity, low cost, and strong photosensitivity [33,34]. As an n-type semiconductor with a band gap of 3.37 eV, ZnO efficiently degrades organic pollutants under UV light. However, issues such as particle agglomeration in suspension and a non-porous structure—leading to low adsorption capacity—make it difficult to use ZnO for effective photodegradation of organic compounds [33,35]. In addition, semiconductors like TiO_2_, ZnO, and CdS generally have limited spectral range (UV) and may undergo photo-corrosion, contributing to insufficient catalytic performance. Thus, developing robust, efficient, and cost-effective catalysts that respond to visible light remains a pressing challenge [34].

Metal-organic frameworks (MOFs) show promise in this regard [36,37,38,39,40], particularly for photocatalytic applications [41,42,43,44]. Consisting of metal centers and organic linkers, MOFs can combine the functionalities of inorganic and organic components in a single composite [45]. Since the initial use of MOFs as photocatalysts in 2006 [46,47], research into their potential for dye photodegradation has expanded significantly [34,42,48,49,50,51,52,53,54,55,56,57,58,59,60,61]. MOFs offer high stability, large surface areas, and controllable porous structures [46]. For instance, MOF-5, which contains Zn4O clusters and organic ligands that act as light-harvesting antennae, exemplifies these features [61,62].

Recent work has demonstrated the possibility of loading MOFs with semiconducting metal oxides, distributing metal nanoparticles within the MOF matrix to maximize the number of catalytic sites [24,25]. In addition to unsaturated metal sites (Lewis acid sites) [19,20], MOFs can include active functional groups (–NH2 [26,27] -SO_3_H [28]) that further improve photocatalytic performance [63]. Fischer et al. introduced ZnO quantum dots into MOF-5 in 2008 via a “ship-in-the-bottle” method, though the ZnO insertion produced structural defects [64,65]. More recently, ZnO-based porous materials have garnered attention as photocatalysts, such as the 3D graphene network loaded with ZnO nanoparticles reported by Cao et al. [51]. Although these systems degrade methylene blue (MB) within short timeframes (e.g., 60 min under UV), many of them remain UV-dependent because of ZnO’s large band gap.

Moving toward visible-light activation, Zhu et al. prepared MOF-derived ZnO composites with reduced graphene oxide (RGO) via a microwave-assisted method, observing enhanced MB photodegradation under visible light [53]. However, combining ZnO with isoreticular MOFs holds particular promise for further improvements. IRMOF-3, which features high porosity, low cost, and demonstrated catalytic activity in the visible-light region [66,67], has been investigated for multiple applications, including cascade reactions with Pd@IRMOF-3 [66], oxidative desulfurization via Ti-modified IRMOF-3 [68] Suzuki and Stille coupling (Pd–IRMOF-3) [69], and more [70,71,72,73]. Several studies have also explored IRMOF-3 for targeted drug delivery [74], optical sensing [73], and gas adsorption [75,76]. Despite this broad versatility, reports on IRMOF-3 loaded with ZnO for MB oxidation under visible light remain scarce.

Given the global concerns regarding synthetic dye pollution—more than 7 × 10^5^ tons of industrial colorants are produced annually, contributing significantly to water contamination [77]—it becomes imperative to explore effective remediation strategies. Methylene blue (MB), widely used in textiles, microbiological staining, and histological applications, is known for its ecotoxicity and carcinogenic potential [5,78,79,80]. Concentrations as low as 1 mg·L^−1^ produce visibly detectable coloration [77], necessitating careful treatment. Therefore, we sought to leverage IRMOF-3’s visible-light sensitivity alongside ZnO’s known photocatalytic potential to develop a hybrid IRMOF-3/ZnO material for the photodegradation of MB. As ZnO absorbs predominantly in the UV range (only 3–5% of the solar spectrum) [81], pairing it with IRMOF-3 (which captures a larger portion of visible light [82]) could significantly extend its operational range and improve photocatalytic efficiency. This study describes the synthesis of the IRMOF-3/ZnO hybrid, its physicochemical characterization, and its performance in the visible-light-driven degradation of MB, thereby filling a gap in the existing research on integrated MOF–semiconductor catalysts.

## 2. Results and Discussion

### 2.1. SEM Characterization

**Figure 1:**
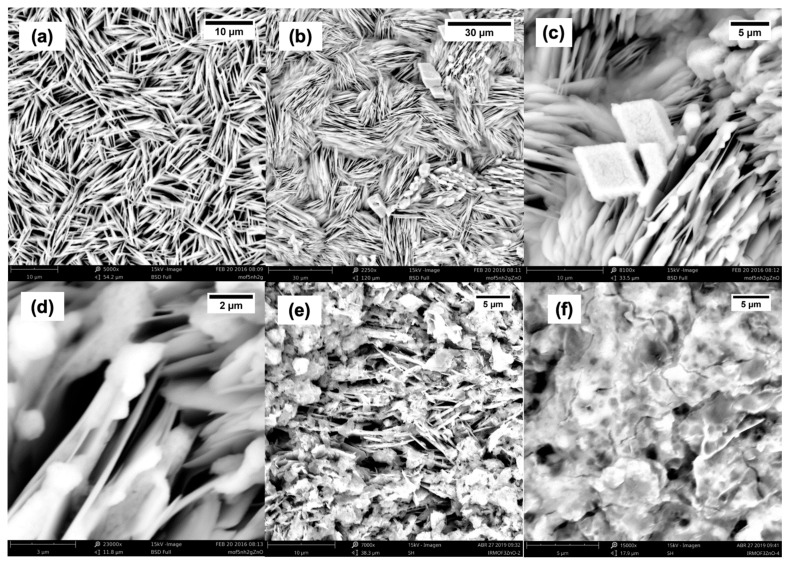
SEM images of (**a**) IRMOF-3 at 5000× magnification; (**b**–**d**) IRMOF-3/ZnO at 2250×, 8100×, and 23,000× magnifications, respectively; (**e**) IRMOF-3/ZnO(2) at 7000× magnification; and (**f**) IRMOF-3/ZnO(4) at 15,000× magnification.

Figure 1 shows SEM images of IRMOF-3, IRMOF-3/ZnO, IRMOF-3/ZnO(2), and IRMOF-3/ZnO(4). Figure 1a shows the SEM image of IRMOF-3 at 5000× magnification. Zheng et al. reported that images with a scale bar of 10 μm best illustrate the relative sizes of the interleaved sheets with elongated rhombus forms [83]. Figure 1b, Figure 1c, and Figure 1d show the SEM images of IRMOF-3 after functionalization with ZnO particles at 2200×, 81,000× and 230,000× magnifications, having scale bars of 30, 5, and 2 μm, respectively. The elongated diamond-shaped intercalated sheets are identified as IRMOF-3. However, particles grown on the film surfaces and between the sheets are observed after functionalization with ZnO. Hence, ZnO particles are introduced and grow inside the IRMOF-3 pores while other ZnO particles grow on the surface, confirming the functionalization of IRMOF-3 materials with ZnO. Figure 1e shows the SEM image of IRMOF-3(2) at 7000× magnification. The pores are filled, unlike those observed in the image of IRMOF-3/ZnO, and excess ZnO is also observed, but it maintains its elongated diamond-shaped morphology. Figure 1f shows an SEM image of IRMOF-3(2) at 15,000× magnification. A continuous surface is observed, and the pores are no longer visible as they are filled with ZnO.

### 2.2. FTIR Characterization

**Figure 2:**
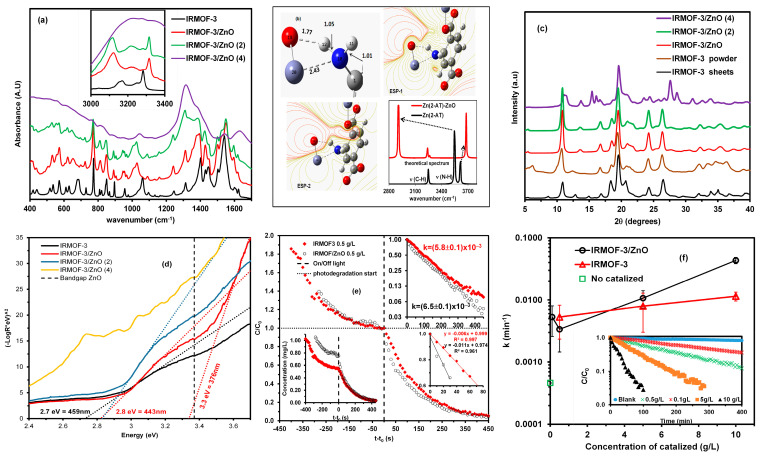
(**a**) FTIR spectra of IRMOF-3 and IRMOF-3/ZnO 400 to 1600 cm^−1^. Inset: Vibrational bands in the spectral range from 3000 to 3400 cm^−1^. (**b**) Theoretical calculations. Illustration of the interaction between Zn (2-AT) and ZnO. ESP (ESP-1 is the ESP in the plane containing atoms 10, 12, and 19; ESP-2 is the same plane with an elevation of 0.5 Å) and theoretical IR spectra for Zn(2-AT) and Zn(2-AT)-ZnO interactions. (**c**) XRD patterns of IRMOF-3, IRMOF-3/ZnO, IRMOF-3/ZnO(2), and IRMOF-3/ZnO(4). (**d**) Band-gap energy comparison for IRMOF-3, IRMOF-3/ZnO, IRMOF-3/ZnO(2), and IRMOF-3/ZnO(4). (**e**) Concentration of MB and IRMOF-3 or IRMOF-3/ZnO as a function of the time in the presence and absence of visible light. (**f**) Apparent kinetic constants for IRMOF-3 and IRMOF-3/ZnO systems with different catalyst concentrations. Inset: dynamics of MB photodegradation under visible light with various concentrations of IRMOF-3/ZnO.

The IR spectra of IRMOF-3, IRMOF-3/ZnO, IRMOF-3/ZnO(2), and IRMOF-3/ZnO(4) were recorded and compared to investigate any band shifts and the appearance or disappearance of vibrational bands, corresponding to bond formation or breakage, respectively, as a means to confirm the formation of a new hybrid material. Figure 2a shows the IR spectrum of IRMOF-3, which is denoted by a solid black trace, and the IR spectra of IRMOF-3/ZnO, IRMOF-3/ZnO(2), and IRMOF-3/ZnO(4), which are indicated by a solid red, green, and violet traces, respectively. Several shifts are confirmed, indicative of the formation of a new hybrid material between IRMOF-3 and ZnO.

A strong red shift of the v(C-N) signal for IRMOF-3 from 1060 to 1030 cm^−1^ is observed. This is due to the weakening of the C-N bond. Furthermore, a small blue shift of 1540 to 1550 cm^−1^ for v(N-H) is observed, which is due to the steric hindrance generated when the zinc is complexed to the amino group nitrogen (Figure 2a). The inset of Figure 2a shows the most important vibrational modes in the IRMOF-3 spectra corresponding to the symmetric and asymmetric v(N–H) stretching at 3160 and 3270 cm^−1^, respectively. When the oxygen of the ZnO material interacts with the hydrogen of the NH_2_ group, N–H bond lengthening occurs since the negative oxygen pulls the positive hydrogen closer to it due to electrostatic interaction. This polarization weakens the N–H bond, generating a redshift of the symmetric v(N–H) stretching band to 3122 cm^−1^ and an increase in intensity.

Furthermore, a blue shift of the asymmetric v(N–H) stretching band to 3317 cm^−1^ and a concurrent decrease in intensity is observed. This indicates a shortened N–H bond. This observation can be explained based on the complexation between the zinc in ZnO and the nitrogen of the NH_2_ group. This N–H bond contraction could also be attributed to the short-range forces faced by H in the complex and to the effect on the electric field of the Zn. Thus, these results confirm generating of a new hybrid material between IRMOF-3 and ZnO. However, the spectrum of IRMOF-3/ZnO(4) is very different from those of IRMOF, IRMOF-3/ZnO, and IRMOF-3/ZnO(2), indicating that loading the MOF four times with ZnO damages its structure.

### 2.3. Theoretical Calculations

Figure 2b (upper left quadrant) illustrates the complexation of ZnO by the amino group in the organic base 2-aminoterephthalic acid (2-AT). The oxygen atom from ZnO forms a hydrogen bond with one of the 2-AT amino group hydrogens, and the zinc atom is complexed with the 2-AT amino group nitrogen. This results in the elongation of the N-H bond of the hydrogen that forms the hydrogen bond and a shortening of the N-H bond of the non-interacting hydrogen. This causes changes in the v(N–H) stretching symmetric and asymmetric mode signals of 2-AT, i.e., a blue shift in the asymmetric mode and a red shift in the symmetrical mode (see Figure 2b, lower right quadrant). This corresponds well with the experimental data.

ESP was calculated in the plane containing the Zn and O of the ZnO and the 2-AT N atom. This was realized to verify the electrostatic environment generated by the partial charges of the atoms and the delocalized electrons (see Figure 2b, upper right quadrant). The ESP generated by the lone electron pair on the nitrogen in 2-AT is very small compared to that generated by the O of ZnO. This suggests some charge displacement from the N to the Zn. Thus, a new ESP in the plane parallel to the previous one displaced by 0.5 Å was generated (see Figure 2b, lower right quadrant) to verify the size of the ESP generated by the nitrogen lone pair, revealing only positive ESP in this new plane, which means that the electron density of the nitrogen lone pair is small. A calculation of the partial charge for the nitrogen in 2-AT before and after complexation shows that it decreases, confirming the above.

### 2.4. XRD Characterization

Figure 2c shows the X-ray diffraction (XRD) patterns of IRMOF-3, IRMOF-3/ZnO, IRMOF-3/ZnO(2), and IRMOF-3/ZnO(4). The XRD patterns reveal that the functionalization of IRMOF-3 with ZnO does not change the structure because the peak positions are not significantly altered. Still, there is a loss of intensity for some of the peaks [84,85] attributable to the IRMOF-3 sheets. Due to multiple factors, some of the characteristic IRMOF-3 reflections diminish or disappear upon ZnO loading. First, pulverizing IRMOF-3 into a fine powder and incorporating ZnO nanoparticles can disturb the original long-range order of the MOF, decreasing its crystallinity and causing specific diffraction peaks to weaken. Second, as ZnO clusters insert into or anchor onto the IRMOF-3 framework, they may generate local defects or slightly modify the lattice geometry, which can further suppress or shift specific reflections. Finally, moisture absorption during sample processing—whether from washing steps or ambient humidity—can also distort the MOF structure, contributing to peak broadening or partial loss of intensity. Together, these effects indicate that although the IRMOF-3 framework remains present, its lattice is partially perturbed or reconfigured due to the presence of ZnO and residual water.

A comparison of the XRD patterns for IRMOF-3/ZnO and unmodified IRMOF-3 reveals a new peak at 13.6°, as shown in Figure 2c. This peak may be attributed to the modification generated by the coordination between ZnO and the amino groups. This is also evidenced in the FT-IR spectrum (see Figure 2a). Moreover, as shown in Figure 2c, the XRD pattern of IRMOF-3/Zn(4) shows many different and new signals. This demonstrates that the structure of the MOF degrades upon repeated heating, as also evidenced by the IR data.

### 2.5. Band-Gap Determination

The band gaps of IRMOF-3, IRMOF-3/ZnO, IRMOF-3/ZnO(2), and IRMOF-3/ZnO(4) in the visible and UV regions were analyzed (Figure 2d). They were calculated by plotting [−log(R)·E]m vs. E (E is in eV), where m = 2. The ZnO band gap is denoted as a black dotted line in the UV region with a value of 3.37 eV [86]. The visible region extends up to 3.1 eV, equivalent to the violet and blue light ranges from 516 to 400 nm. The signals corresponding to IRMOF-3 with a band-gap value of 2.7 eV and IRMOF-3/ZnO and IRMOF-3/ZnO(2), both with a band-gap value of 2.8 eV, are observed in the visible region. In other words, the band gap of IRMOF-3 increases upon loading with ZnO. IRMOF-3 can absorb visible light due to the amine group in the structure [45,87,88,89], which explains the band gap modification when ZnO interacts with the amine group of IRMOF-3. The band gap for IRMOF-3/ZnO(4) cannot be measured due to the degradation of its structure.

### 2.6. Artificial Photoreaction and Adsorption-Photodegradation Measurements

The photoreaction of 0.5 g·L^−1^ IRMOF-3/ZnO and IRMOF-3 in 25 mL of 1 mg/L MB solution under a white-light lamp was monitored. The variation in MB’s maximum absorbance at 670 nm with irradiation time was monitored. This maximum absorbance decreases with time due to the photodegradation of the dye.

A solution of 0.5 g·L^−1^ IRMOF-3/ZnO or IRMOF-3 in the presence of MB was placed in the dark (Figure 2e). Absorbance measurements were performed every 5 min. Initially, the MB concentration slightly decreases with time, and the adsorption capacity is lower for the modified material. This is attributed to the fact that ZnO occupies pores, as is evident from the SEM images (Figure 1). The sample was placed under a white-light lamp when the concentration reached the adsorption equilibrium value. Photodegradation was observed (Figure 2e), where the onset of photodegradation is identified with the dashed line. Light irradiation leads to a rapid decrease in MB concentration.

IRMOF-3/ZnO and IRMOF-3 utilize two mechanisms for decontaminating MB-containing water, i.e., material adsorption and photodegradation, which occur synergistically. Although the adsorption capacity of the modified material is lower than that of the pristine MOF, it is still significant. This experiment was designed to demonstrate that the photodegradation mechanism occurs and that not only is the contaminant adsorbed onto the material. The photodegradation capacity of the material is of greater importance because it converts the contaminant to simpler molecules, and the material can then be reused. The adsorption mechanism relates to the material’s porosity and high surface area. In the adsorption experiment, IRMOF-3 presents a higher adsorption capacity than IRMOF-3/ZnO (Figure 2e, lower left quadrant). This is to be expected because ZnO partially occupies the pores in IRMOF-3/ZnO. However, in the photodegradation experiment, the IRMOF-3/ZnO effects greater photodegradation than IRMOF-3 (Figure 2e, upper right quadrant). The photodegradation curves fit a first-order exponential, with the rate constant for IRMOF-3/ZnO being higher than that of IRMOF-3. A more detailed analysis of the kinetic behavior at the beginning of the photodegradation process revealed that the rate over the hybrid material is double that over the pristine MOF (Figure 2e, lower right quadrant). This demonstrates the improvement in photodegradation by including ZnO in the IRMOF-3.

### 2.7. Effect of Catalyst Concentration

The dynamics of MB concentration for various catalyst loadings were monitored. Figure 2f, inset shows the results obtained for 10 g·L^−1^ of the catalyst (black triangles), 5 g·L^−1^ of the catalyst (orange squares), 0.5 g·L^−1^ of the catalyst (green crosses), 0.1 g·L^−1^ of the catalyst (red; x), and a blank sample (light blue dots). For each run, the initial MB concentration was 1 mg·L^−1^.

The speed of photodegradation is dependent on the concentration of the catalyst (Figure 2f), and the most rapid dye degradation is observed using 10 g·L^−1^ of the hybrid material (Figure 2f, insert).

Furthermore, the concentration of MB in the blank reaction decreases to a small extent (Figure 2f, insert) due to the natural photodegradation of the dye without the catalyst. However, this photodegradation is very slow, confirming the necessity of a catalyst to achieve the efficient degradation of MB. Table 1 summarizes the effect of catalyst concentration on the degradation rate constant and reaction half-life. The apparent first-order rate constants for different catalyst concentrations were calculated using Equation (4), and the half-life for each reaction was calculated using Ln(2)/kc.

Equation (1) was derived as follows:

(1) $$-\frac{d\left[A\right]}{dt}=k\left[A\right]$$

where k = k_c S; k is the apparent kinetic constant; k_c is the kinetic constant; [A] is the MB concentration; and S is the surface area. Solving for [A](t),

(2) $$\left[A\right]=\left[A_{0}\right]e^{-kt},$$

To compare the effect of catalyst concentration on the reaction rate with that for other visible-region MB photodegradation catalysts, the same analysis was performed for the IRMOF-3 catalyst in the same range of concentrations used for the hybrid material. A relationship was observed between the apparent kinetic constants and the different catalyst concentrations used for IRMOF-3 and IRMOF-3/ZnO.

The black points in Figure 2f correspond to the hybrid material. Furthermore, the catalyst concentration is effectively proportional to the reaction kinetics, confirming that an increase in catalyst concentration leads to more rapid MB degradation. However, the behavior of IRMOF-3 (denoted by red triangles) demonstrates that the hybrid catalyst is more efficient than IRMOF-3, further confirming that functionalization highlights the individual material characteristics.

### 2.8. Catalyst Reuse Capacity

**Figure 3:**
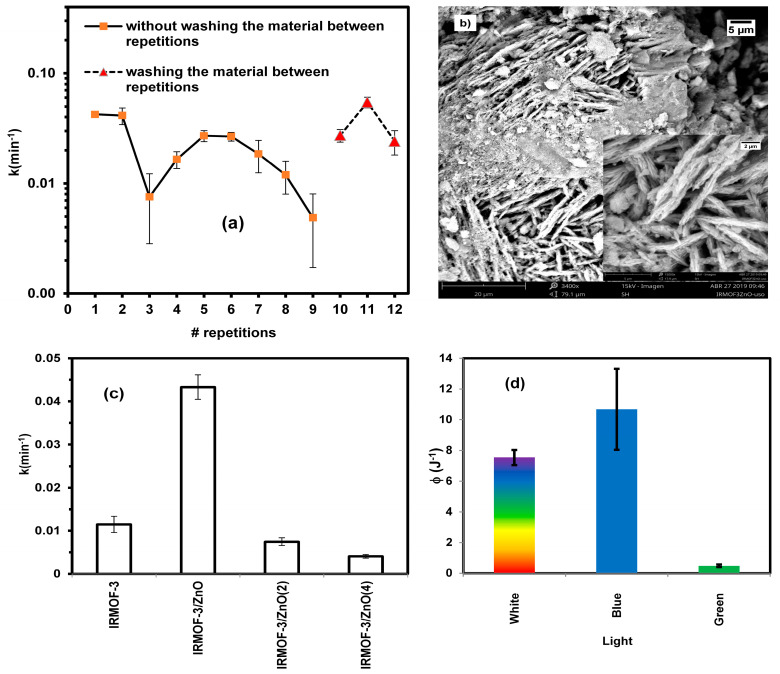
(**a**) Effect of material reuse on photocatalytic activity. (**b**) SEM images of IRMOF-3/ZnO after 11 uses for the degradation of MB. (**c**) Effect of multiple impregnations of IRMOF with ZnO on reaction kinetics. (**d**) Reaction efficiencies under white, blue, and green light.

The reuse of 10 g·L^−1^ of IRMOF-3/ZnO was examined using the same material 11 times. Figure 3a shows the apparent kinetic constants for the hybrid material with respect to the number of repetitions. A total of 11 repetitions were performed over three days; two repetitions on the 1st and the 2nd days (denoted by orange squares) and three repetitions on the 3rd day (indicated by red triangles). On the last day, the material was rinsed with water. The material effectively degrades MB in each run (Figure 3a), demonstrating that the hybrid material can be reused for the degradation of MB.

The material’s behavior after rinsing with water should also be considered, as the rate constant is higher on the 3rd test day (Figure 3a). Thus, the material should be washed after each photodegradation runs to maintain efficiency and activity.

Figure 3b shows an SEM image of IRMOF-3/ZnO at 3400× magnification after 11 repetitions. The material’s structure remains original, and some deposits and degradation are observed. These may be due to degradation processes of itself and/or waste from the photodegradation process analyte. However, the material can be stable to reuse under the conditions employed in this study. Still, it is possible that real ambient where other substances exist in the medium is not as efficient.

### 2.9. Effect of Multiple Impregnations of IRMOF with ZnO

Figure 3c shows a histogram representation of the rate constants for IRMOF-3, IRMOF-3/ZnO, IRMOF-3/ZnO(2), and IRMOF-3/ZnO(4). IRMOF-3/ZnO(2) and IRMOF-3/ZnO(4) were prepared to determine whether increased material saturation with ZnO improves degradation efficiency.

The degradation rates over IRMOF-3/ZnO(2) and IRMOF-3/ZnO(4) are considerably lower than that over IRMOF-3/ZnO and, surprisingly, even that over IRMOF-3 alone. Thus, the oversaturation of IRMOF-3 is counterproductive as the material quality deteriorates, most likely due to blocking the pores in IRMOF-3, leading to a dramatic decrease in surface area and, subsequently, MB adsorption ability and degradation efficiency.

### 2.10. Dependence of Photocatalytic Activity on Light Energy

The white source was filtered using a blue filter (440–500 nm) or a green filter (500–600 nm), and photodegradation experiments were performed using these different filters. The efficiency was calculated from the irradiance and rate constant for each filter and the all-white source. The hybrid material efficiently promotes dye photodegradation under blue light (Figure 3d). This result is consistent with the band-gap measurements.

### 2.11. Comparison with Other Visible-Light Photocatalytic Systems

The photocatalytic performance of hybrid IRMOF-3/ZnO can be contextualized by comparing it with various doped or sensitized semiconductor materials reported in the literature. One notable approach involves cobalt doping of ZnS to narrow the band gap and thereby extend light absorption into the visible range. In such Co-doped ZnS systems, increasing dopant levels systematically red-shifts the band gap from approximately 3.3 eV to 2.65 eV, considerably boosting the photodegradation rate of methylene blue (MB) under simulated solar irradiation (Wang et al. [29]). Although these metal-ion doping strategies can significantly improve visible-light harvesting, very high dopant concentrations sometimes lead to structural defects or decreased crystallinity. In our IRMOF-3/ZnO composite, the amine-containing organic linkers of IRMOF-3 play a similar role in shifting the effective band gap to around 2.8 eV, yet do so without external metal dopants. In addition, the porous nature of the MOF framework can further enhance dye adsorption and transport, potentially balancing reactivity gains with long-term stability.

A second avenue for visible-light-driven catalysis appears in dye-sensitized TiO_2_ materials, where organic dyes (e.g., porphyrins, chlorin e6) extend TiO_2_ absorption into the visible region through a “light antenna” effect (Youssef et al. [26]). Although these systems generally display remarkable improvements in photodegradation of model pollutants such as methylene blue or methyl orange, the dye molecules can degrade or detach from the catalyst surface over multiple runs, reducing overall stability. By contrast, the built-in organic linkers in IRMOF-3 mean that our IRMOF-3/ZnO catalyst does not rely on adsorbed dyes; instead, the framework itself is responsible for absorbing visible light. This embedded sensitizer strategy not only provides a simpler route to visible-light activation but also ensures that the light-harvesting moiety remains stably integrated in the hybrid material.

Another promising route exploits silver-decorated ZnO architectures to enhance photocatalytic efficiency and even confer antibacterial properties (Lam et al. [27]). Here, noble-metal nanoparticles (Ag) serve as electron sinks, suppressing electron–hole recombination and enabling superior dye removal. Despite the success of such noble-metal modifications, potential issues related to material costs and metal-ion release may arise in long-term applications. In comparison, coupling ZnO with IRMOF-3 leverages the well-known photocatalytic activity of ZnO while taking advantage of the MOF’s high surface area and amine-driven visible-light response. This synergy offers an alternative for water treatment processes that does not require the inclusion of expensive metals.

Beyond MB degradation, similar photocatalysts have targeted various dyes or even different types of organic contaminants (e.g., crystal violet, fast green, ibuprofen) through doping with non-metals or fabricating core–shell/hollow structures (Kim et al. [25]; Khedr et al. [28]). Although specific details, such as pollutant molecules or reactor geometries, differ among these studies, the overarching goal remains the same: boosting the material’s visible-light absorption and promoting fast, efficient photodegradation. The IRMOF-3/ZnO catalyst achieves both objectives by unifying ZnO’s robust catalytic features with IRMOF-3’s extended absorption and pore-rich environment, yielding effective MB mineralization under mild conditions and straightforward syntheses. Hence, while doping, noble-metal deposition, or organic dye-sensitization have each proven effective for visible-light photocatalysis, merging IRMOF-3 with ZnO distinctly combines stable visible-light harvesting and intrinsic adsorption capabilities in a single hybrid platform.

## 3. Materials and Methods

### 3.1. IRMOF-3 Synthesis

IRMOF-3 was synthesized via a solvothermal method [68,85,90,91,92,93] using 2-aminoterephthalic acid (2-AT) (Sigma-Aldrich, Milwaukee, WI, USA) and zinc nitrate hexahydrate (Zn(NO_3_)_2_·6H_2_O (Alfa Aesar, Haverhill, MA, USA) at a ratio of 1:4. First, a solution of 1.234 g of Zn(NO_3_)_2_·6H_2_O and 0.3 g of 2-AT in 10 mL of dimethylformamide (DMF) (Alfa Aesar) was sealed and heated in an oven for 24 h at 105 °C. Then, the material was washed with DMF twice after a methanol wash and finally with dichloromethane. This was allowed to dry in a vacuum at 40 °C.

### 3.2. ZnO-Functionalized IRMOF-3

First, a Zn(NO_3_)_2_·6H_2_O-saturated solution was prepared, and 10 mL of this solution was added to 1 g of IRMOF-3 sample. Then, the solution was left at room temperature for 24 h. Next, the material was washed twice with distilled water, rinsed with acetone to remove excess zinc nitrate, and placed in a tubular oven at 90 °C for 24 h before cooling to room temperature. The as-obtained hybrid material (IRMOF-3/ZnO) was powdered, sealed, and stored in the dark.

Two other composites were prepared by impregnating IRMOF-3 with ZnO two and four times. A 10-mL aliquot of the saturated Zn(NO_3_)_2_·6H_2_O solution was added to 1 g of IRMOF-3 sample. The solution was left at room temperature for 24 h. Then, the material was washed twice with distilled water, rinsed with acetone to remove excess zinc nitrate, transferred to an oven, and heated at 105 °C for 24 h. For the second impregnation, the material was submerged in the saturated Zn(NO_3_)_2_·6H_2_O solution for 24 h and rinsed twice with water and twice with acetone. The sample was then transferred into an oven and heated at 105 °C for 12 h. The abovementioned process was repeated twice, affording a sample impregnated four times. Finally, the sample was sealed and kept in the dark. The IRMOF-3 impregnated twice with ZnO is labeled IRMOF-3/ZnO(2), and that impregnated four times is labeled IRMOF-3/ZnO(4).

### 3.3. Catalyst Characterization

The materials were characterized using several methods. The morphologies were observed using scanning electron microscopy (SEM; Phenom Pro X, Phenom-World, Eindhoven, The Netherlands). X-ray diffraction (XRD) patterns were recorded using a Thermo Scientific X-ray diffractometer (Waltham, MA, USA) equipped with a Co-filtered CoKα radiation source. The presence of functional groups and identification of modified bonds and new bonds were investigated using Fourier-transform infrared (FTIR) spectroscopy (ALPHA FTIR spectrometer equipped with an ATR diamond crystal, Bruker Optics, Billerica, MA, USA). Optical band-gap energies were determined using diffuse-reflectance UV-Vis spectrophotometry on an SD2000 spectrometer with a DH-2000 light source coupled to a QR600-7-UV-125F diffuse-reflectance fiber optics probe (Ocean Optics, Largo, FL, USA). Band-gap energies were determined using Equation (3):

(3) $$Eg=\frac{hc}{\lambda }=\frac{1240}{\lambda }eV$$

where Eg is the band-gap energy in eV; h is the Planck constant; c is the speed of light; and λ is the wavelength. First, the UV-Vis spectrophotometer was used to measure the reflectance of MOF5, IRMOF-3, and IRMOF-3/ZnO. Second, using Equation (3), the conversion from nanometers to electron volts was performed. Then, the negative value of the logarithm of the reflectance [−log(R)] was obtained for each material and multiplied by the energy (eV). The results were then squared. Finally, the band gap of each material was obtained by plotting [−logR·E]m vs. E (eV) using the direct method, where m = 2 [7].

### 3.4. Metodology for Adsorption-Photodegradation Measurements

Several suspensions of IRMOF-3 or the hybrid IRMOF-3/ZnO (0 to 10 g·L^−1^) were prepared in clear glass vials, each containing 25 mL of mg·L^−1^ methylene blue (MB). The vials were placed under a white-light-emitting diode (LED) lamp (1200 lumen, 110 V, 60 Hz, 15 W, Ilumax) until the dye was completely degraded. A control experiment (no catalyst) was also performed to track MB degradation under the same conditions. The photoreaction time varied from 2 to 10 h, depending on the catalyst concentration.

To ensure that predominantly visible wavelengths reached the sample, a long pass colored glass filter with a cutoff at 400 nm was positioned between the top-illuminating LED lamp and the reaction vessel. The vessel was wrapped in reflective aluminum foil to enhance light utilization, and a magnetic stirrer was employed to maintain a uniform suspension of the catalyst throughout the experiment.

During the reaction, a 2-mL aliquot of the solution was withdrawn every 5 min, placed in a quartz cell, and analyzed using a UV-Vis spectrophotometer. A calibration curve covering 0–1 mg·L^−1^ was used to determine the MB concentration (detection limit 0.04 mg·L^−1^, R^2^ = 0.998). Each aliquot was returned to the reaction vessel immediately after measurement to maintain the total reaction volume. Additionally, the samples were left for a further 48 h under white-light illumination to assess the absorbance at infinite time (i.e., post-complete degradation).

### 3.5. Effect of Catalyst Concentration Methodology

Several concentrations of IRMOF-3/ZnO (i.e., 0.1, 0.5, 5, and 10 g·L^−1^), IRMOF-3 (i.e., 0.1, 0.5, 5, and 10 g·L^−1^) and a blank (no catalyst) with 25 mL of 1 g·L^−1^ MB solution were investigated. The methodology corresponded to that used for the aforementioned artificial photoreaction. Tests were repeated twice to reduce bias in the result.

### 3.6. Catalyst Recyclability

This experiment determined whether the prepared hybrid catalyst could be recycled. For this purpose, 10 g·L^−1^ of IRMOF-3/ZnO was prepared. Then, the sample was placed into a glass bottle with 25 mL of MB solution (1 mg·L^−1^) under white-light irradiation. Then, absorbance spectra were recorded every 5 min until the MB had been successfully degraded (typically 60 min), and the kinetic parameters were calculated. Subsequently, eight photodegradation experiments were performed using the same material without washing the material between repetitions, and three photodegradation experiments were performed with the washing of the materials between repetitions.

### 3.7. Effect of Multiple ZnO Impregnations

A test was conducted to investigate how repeated impregnation of IRMOF-3 with the ZnO semiconductor affects the kinetic constant and material efficiency with respect to MB degradation. For this test, 10 g·L^−1^ of each catalyst, i.e., IRMOF-3, IRMOF-3/ZnO, IRMOF-3/ZnO(2), and IRMOF-3/ZnO(4), was used for the photodegradation of 1 mg·L^−1^ MB solutions under visible-light exposure in glass vials. The photodegradation and kinetic analyses were performed the same manner as those above.

### 3.8. Effect of Light Energy on Photodegradation Efficiency

Emissions from different light sources (i.e., white lamp, 440–500-nm blue filter, and 500–600-nm green filter) were measured using a UV-Vis spectrophotometer (Ocean Optics, Largo, FL, USA) calibrated in units of μW/cm^2^·nm. Hence, the specific visible-light range within which photodegradation occurred was identified. To measure the power of each source used, the area under the emission spectra curve (μW/cm^2^) of the source was calculated and multiplied by the area of the catalyst illuminated in cm^2^ (μW).

Since the kinetics constant k depends on the amount of light, it was divided by the irradiance (I; W), which was previously obtained (Equation (4)):

(4) $$\phi =\frac{k}{I}$$

where ϕ is a parameter that measures the efficiency of the type of light, which does not depend on the light intensity. This efficiency allows us to identify the specific visible-light range in which the hybrid material takes optimal advantage of incident energy.

### 3.9. Theoretical Calculations Methodology 

The structure of the organic base 2-AT with one Zn and the interaction of ZnO with the amino group of 2-AT were investigated by Density Functional Theory (DFT) method at the B3LYP/L level of theory using Gaussian 09W [94]. Vibrational spectra and electrostatic surface potentials (ESP) were calculated to investigate the interaction of ZnO with IRMOF-3.

## 4. Conclusions

This study has demonstrated that combining IRMOF-3 with ZnO yields a hybrid material (IRMOF-3/ZnO) capable of efficiently adsorbing and degrading methylene blue (MB) under visible-light irradiation. The synergy of these two components provides both an adsorption mechanism—enhanced by the high porosity of IRMOF-3—and a photodegradation mechanism, driven by ZnO’s semiconducting properties. Compared to pristine IRMOF-3, the hybrid exhibited superior performance by exploiting two distinct energy-transfer pathways: (1) extra-molecular transfer from the IRMOF-3 amine linkers, which act as visible-light antennas, to the ZnO, and (2) intra-molecular transfer from Zn atoms back into the framework. This dual radical-generation route increases overall degradation efficiency.

A key advantage of the IRMOF-3/ZnO hybrid is its ability to operate under visible light (band gap of approximately 2.8 eV), which aligns well with the solar spectrum. Consequently, this material can harness a readily available and sustainable energy source—sunlight—to degrade organic pollutants. Such a feature makes it an appealing alternative to conventional UV-dependent photocatalysts like TiO_2_ or ZnO alone, enabling broader applicability in water treatment settings.

Despite these benefits, certain drawbacks should be considered when moving toward large-scale implementation. First, the IRMOF-3 framework may experience partial structural degradation if subjected to extreme thermal or chemical conditions, necessitating careful control of reaction parameters. Second, although ZnO incorporation improves the material’s optical response, uniform and reproducible loading of ZnO within the MOF can be challenging, and overloading may reduce porosity. Finally, initial fabrication and handling costs of the MOF component could be higher than those of standard inorganic catalysts. Addressing these limitations—through optimized synthesis protocols, thorough stability assessments, and cost-effectiveness studies—will be vital for fully realizing the promise of IRMOF-3/ZnO in practical water-purification applications.

## Figures and Tables

**Table 1:** Effect of IRMOF-3/ZnO catalyst concentrations on kinetic parameters.

	IRMOF-3/ZnO		IRMOF-3	
Concentrations (g*L^−1^)	k (min^−1^)	Half-Life Time (min)	k (min^−1^)	Half-Life Time (min)
10.0	0.043 ± 0.003	16	0.011 ± 0.002	60
5.0	0.011 ± 0.003	65	0.008 ± 0.005	87
0.5	0.003 ± 0.002	206	0.005 ± 0.003	131
0.1	0.005 ± 0.003	131		
0.0	0.00047 ± 0.00001	1490		

## Data Availability

All data supporting the findings of this study are included in the article.

## Abbreviations

The following abbreviations are used in this manuscript:

MDPI	Multidisciplinary Digital Publishing Institute
MOFs	Metal-organic frameworks
IRMOF-3	basic isoreticular metal-organic framework
3DGN	3D graphene network
DFT	Density Functional Theory
MB	methylene blue
2-AT	2-aminoterephthalic acid
XRD	X-ray diffraction
ESP	electrostatic surface potentials

## References

[1] UNESCO (2003). Agua Para Todos, Agua Para la Vida.

[2] Gajbhiye S.B. (2012). Photocatalytic degradation study of methylene blue solutions and its application to dye industry effluent. Int. J. Mod. Eng. Res..

[3] Hassena H. (2016). Photocatalytic Degradation of Methylene Blue. Mod. Chem. Appl..

[4] Cortazar A., Coronal C., Escalante A., Gonzalez R. (2010). Contaminación generada por colorantes de la industria textil. Univ. Autón. Estado Hidalgo.

[5] Moreno A., Figueroa D., Hormaza A. (2012). Adsorption of methylene blue on rice hulls. Prod. Limpia.

[6] Arroyave Rojas J.A., Rodríguez Gaviria E.M., Barón Aristizábal C.A., Moreno Salazar C.C. (2012). Degradación y mineralización del colorante rojo punzó empleando el reactivo de Fenton. Prod. Limpia.

[7] Souza R.P., Freitas T.K., Domingues F.S., Pezoti O., Ambrosio E., Ferrari-Lima A.M., Garcia J.C. (2016). Photocatalytic activity of Ti_2_, ZnO and Nb_2_O_5_ applied to degradation of textile wastewater. J. Photochem. Photobiol. A Chem..

[8] Uher G., Pillans J.J., Hatton A.D., Upstill-Goddard R.C. (2017). Photochemical oxidation of dimethylsulphide to dimethylsulphoxide in estuarine and coastal waters. Chemosphere.

[9] Shankaraiah G., Saritha P., Bhagawan D., Himabindu V., Vidyavathi S. (2017). Photochemical oxidation of antibiotic gemifloxacin in aqueous solutions—A comparative study. S. Afr. J. Chem. Eng..

[10] Akyol A., Can O.T., Bayramoglu M. (2015). Treatment of hydroquinone by photochemical oxidation and electrocoagulation combined process. J. Water Process Eng..

[11] Ge S., Xu Y., Jia L. (2017). Effects of inorganic seeds on secondary organic aerosol formation from photochemical oxidation of acetone in a chamber. Atmos. Environ..

[12] López-Martín Á., Caballero A., Colón G. (2017). Photochemical methane partial oxidation to methanol assisted by H_2_O_2_. J. Photochem. Photobiol. A Chem..

[13] Sato Y., Takizawa S.-Y., Murata S. (2016). Photochemical water oxidation system using ruthenium catalysts embedded into vesicle membranes. J. Photochem. Photobiol. A Chem..

[14] Lourenço S.D.M., de Oliveira M.G. (2017). Topical photochemical nitric oxide release from porous poly(vinyl alcohol) membrane for visible light modulation of dermal vasodilation. J. Photochem. Photobiol. A Chem..

[15] Liu M., Wan Y., Wang Y., Xu J., Li X. (2024). Robust photoelectrocatalytic degradation of antibiotics by organic-inorganic PDISA/Bi_2_WO_6_ S-scheme heterojunction membrane. J. Environ. Chem. Eng..

[16] Liu M., Ye Y., Xu L., Gao T., Zhong A., Song Z. (2023). Recent Advances in Nanoscale Zero-Valent Iron (nZVI)-Based Advanced Oxidation Processes (AOPs): Applications, Mechanisms, and Future Prospects. Nanomaterials.

[17] Liu M., Ye Y., Ye J., Gao T., Wang D., Chen G., Song Z. (2023). Recent Advances of Magnetite (Fe_3_O_4_)-Based Magnetic Materials in Catalytic Applications. Magnetochemistry.

[18] Parra S., Olivero J., Pacheco L., Pulgarin C. (2003). Structural properties and photoreactivity relationships of substituted phenols in TiO_2_ suspensions. Appl. Catal. B Environ..

[19] APA (2013). Aplicación de la Fotocatálisis Solar a la Degradación de Contaminantes Orgánicos con Catalizadores Nanoestructurados de TiO_2_.

[20] Calvo M.J., Navarro C., Durán P., Galan-Freyle N.J., Parra Hernández L.A., Pacheco-Londoño L.C., Castelanich D., Bermúdez V., Chacin M. (2024). Antioxidants in Photoaging: From Molecular Insights to Clinical Applications. Int. J. Mol. Sci..

[21] Khatamian M., Daneshvar N., Sabaee S. (2010). Heterogeneos Photocatalytic Decolorization of Brown NG by TiO_2_–UV Process. Iran. J. Chem. Chem. Eng..

[22] Mehrabadi Z., Faghihian H. (2018). Comparative photocatalytic performance of TiO_2_ supported on clinoptilolite and TiO_2_/Salicylaldehyde-NH2-MIL-101(Cr) for degradation of pharmaceutical pollutant atenolol under UV and visible irradiations. J. Photochem. Photobiol. A Chem..

[23] Boutiti A., Zouaghi R., Bendjabeur S.E., Guittonneau S., Sehili T. (2017). Photodegradation of 1-hexyl-3-methylimidazolium by UV/H_2_O_2_ and UV/TiO_2_: Influence of pH and chloride. J. Photochem. Photobiol. A Chem..

[24] Gatica E., Natera J., Pajares A., Gambetta C., Sancho M.I., Massad W.A., García N.A. (2017). Cyclodextrine-nanoencapsulation of niclosamide: Water solubility and meaningful enhancement of visible-light—Mediated sensitized photodegradation of the drug. J. Photochem. Photobiol. A Chem..

[25] Kim K.N., Jung H.-R., Lee W.-J. (2016). Hollow cobalt ferrite–polyaniline nanofibers as magnetically separable visible-light photocatalyst for photodegradation of methyl orange. J. Photochem. Photobiol. A Chem..

[26] Youssef Z., Arnoux P., Colombeau L., Toufaily J., Hamieh T., Frochot C., Roques-Carmes T. (2018). Comparison of two procedures for the design of dye-sensitized nanoparticles targeting photocatalytic water purification under solar and visible light. J. Photochem. Photobiol. A Chem..

[27] Lam S.-M., Quek J.-A., Sin J.-C. (2018). Mechanistic investigation of visible light responsive Ag/ZnO micro/nanoflowers for enhanced photocatalytic performance and antibacterial activity. J. Photochem. Photobiol. A Chem..

[28] Khedr T.M., El-Sheikh S.M., Hakki A., Ismail A.A., Badawy W.A., Bahnemann D.W. (2017). Highly active non-metals doped mixed-phase TiO_2_ for photocatalytic oxidation of ibuprofen under visible light. J. Photochem. Photobiol. A Chem..

[29] Wang R., Liang H., Hong J., Wang Z. (2016). Hydrothermal synthesis of cobalt-doped ZnS for efficient photodegradation of methylene blue. J. Photochem. Photobiol. A Chem..

[30] Zayadi R.A., Bakar F.A. (2017). Comparative study on the performance of Au/F-TiO_2_ photocatalyst synthesized from Zamzam water and distilled water under blue light irradiation. J. Photochem. Photobiol. A Chem..

[31] Liang J.-Y., Yuann J.-M.P., Hsie Z.-J., Huang S.-T., Chen C.-C. (2017). Blue light induced free radicals from riboflavin in degradation of crystal violet by microbial viability evaluation. J. Photochem. Photobiol. B Biol..

[32] Grzechulska J., Morawski A.W. (2002). Photocatalytic decomposition of azo-dye acid black 1 in water over modified titanium dioxide. Appl. Catal. B Environ..

[33] Ökte A.N., Karamanis D., Chalkia E., Tuncel D. (2017). The effect of ZnO or TiO_2_ loaded nanoparticles on the adsorption and photocatalytic performance of Cu-BTC and ZIF-8 MOFs. Mater. Chem. Phys..

[34] Chen Y.-Z., Zhang R., Jiao L., Jiang H.-L. (2018). Metal–organic framework-derived porous materials for catalysis. Coord. Chem. Rev..

[35] Buasakun J., Srilaoong P., Chaloeipote G., Rattanakram R., Wongchoosuk C., Duangthongyou T. (2020). Synergistic effect of ZnO/ZIF8 heterostructure material in photodegradation of methylene blue and volatile organic compounds with sensor operating at room temperature. J. Solid State Chem..

[36] Li M.-T., Sun Y., Zhao K.-S., Wang Z., Wang X.-L., Su Z.-M., Xie H.-M. (2016). Metal–Organic Framework with Aromatic Rings Tentacles: High Sulfur Storage in Li–S Batteries and Efficient Benzene Homologues Distinction. ACS Appl. Mater. Interfaces.

[37] Kreno L.E., Leong K., Farha O.K., Allendorf M., Van Duyne R.P., Hupp J.T. (2012). Metal–Organic Framework Materials as Chemical Sensors. Chem. Rev..

[38] Kim K.-J., Lu P., Culp J.T., Ohodnicki P.R. (2018). Metal-Organic Framework Thin Film Coated Optical Fiber Sensors: A Novel Waveguide-Based Chemical Sensing Platform. ACS Sens..

[39] Cui Y., Li B., He H., Zhou W., Chen B., Qian G. (2016). Metal–Organic Frameworks as Platforms for Functional Materials. Acc. Chem. Res..

[40] Guerrero-Medina J., Mass-González G., Pacheco-Londoño L., Hernández-Rivera S.P., Fu R., Hernández-Maldonado A.J. (2015). Long and local range structural changes in M[(bdc)(ted)0.5] (M = Zn, Ni or Cu) metal organic frameworks upon spontaneous thermal dispersion of LiCl and adsorption of carbon dioxide. Microp. Mesoporous Mater..

[41] Fang Y., Ma Y., Zheng M., Yang P., Asiri A.M., Wang X. (2018). Metal–organic frameworks for solar energy conversion by photoredox catalysis. Coord. Chem. Rev..

[42] Wu W., Luo Z.-D., Wang J., Liu J. (2017). Photocatalytic degradation of methyl violet and rhodamine B based on an extremely stable metal-organic framework decorated with carboxylate groups. Inorg. Chem. Commun..

[43] Ramezanalizadeh H., Manteghi F. (2017). Immobilization of mixed cobalt/nickel metal-organic framework on a magnetic BiFeO_3_: A highly efficient separable photocatalyst for degradation of water pollutions. J. Photochem. Photobiol. A Chem..

[44] Yang L., Yu Y., Feng J., Wu J., Jiang L., Dan Y., Qiu Y. (2018). Charge transfer induced unexpected red-shift absorption of Zn and Cu porous coordination polymers based on electron-withdrawing ligand. J. Photochem. Photobiol. A Chem..

[45] Horiuchi Y., Toyao T., Saito M., Mochizuki K., Iwata M., Higashimura H., Anpo M., Matsuoka M. (2012). Visible-Light-Promoted Photocatalytic Hydrogen Production by Using an Amino-Functionalized Ti(IV) Metal–Organic Framework. J. Phys. Chem. C.

[46] Qiu J., Zhang X., Xie K., Zhang X.-F., Feng Y., Jia M., Yao J. (2019). Noble metal nanoparticle-functionalized Zr-metal organic frameworks with excellent photocatalytic performance. J. Colloid Interface Sci..

[47] Mahata P., Madras G., Natarajan S. (2006). Novel photocatalysts for the decomposition of organic dyes based on metal-organic framework compounds. J. Phys. Chem. B.

[48] Wang C.-C., Li J.-R., Lv X.-L., Zhang Y.-Q., Guo G. (2014). Photocatalytic organic pollutants degradation in metal–organic frameworks. Energy Environ. Sci..

[49] Salehifar N., Zarghami Z., Ramezani M. (2016). A facile, novel and low-temperature synthesis of MgO nanorods via thermal decomposition using new starting reagent and its photocatalytic activity evaluation. Mater. Lett..

[50] Liang P., Zhang C., Sun H., Liu S., Tadé M., Wang S. (2016). Photocatalysis of C, N-doped ZnO derived from ZIF-8 for dye degradation and water oxidation. RSC Adv..

[51] Cao X., Zheng B., Rui X., Shi W., Yan Q., Zhang H. (2014). Metal oxide-coated three-dimensional graphene prepared by the use of metal-organic frameworks as precursors. Angew. Chem. (Int. Ed. Engl.).

[52] Chen H., Shen K., Chen J., Chen X., Li Y. (2017). Hollow-ZIF-templated formation of a ZnO@C–N–Co core–shell nanostructure for highly efficient pollutant photodegradation. J. Mater. Chem. A.

[53] Zhu G., Li X., Wang H., Zhang L. (2017). Microwave assisted synthesis of reduced graphene oxide incorporated MOF-derived ZnO composites for photocatalytic application. Catal. Commun..

[54] Andrew Lin K.-Y., Hsu F.-K. (2015). Magnetic iron/carbon nanorods derived from a metal organic framework as an efficient heterogeneous catalyst for the chemical oxidation process in water. RSC Adv..

[55] Zhang C., Ye F., Shen S., Xiong Y., Su L., Zhao S. (2015). From metal–organic frameworks to magnetic nanostructured porous carbon composites: Towards highly efficient dye removal and degradation. RSC Adv..

[56] Xu Q., Guo Z., Zhang M., Hu Z., Qian Y., Zhao D. (2016). Highly efficient photocatalysts by pyrolyzing a Zn–Ti heterometallic metal–organic framework. CrystEngComm.

[57] Ahmed A., Forster M., Jin J., Myers P., Zhang H. (2015). Tuning Morphology of Nanostructured ZIF-8 on Silica Microspheres and Applications in Liquid Chromatography and Dye Degradation. ACS Appl. Mater. Interfaces.

[58] Guo Z., Cheng J.K., Hu Z., Zhang M., Xu Q., Kang Z., Zhao D. (2014). Metal-organic frameworks (MOFs) as precursors towards TiO_x_/C composites for photodegradation of organic dye. RSC Adv..

[59] Yang S.J., Im J.H., Kim T., Lee K., Park C.R. (2011). MOF-derived ZnO and ZnO@C composites with high photocatalytic activity and adsorption capacity. J. Hazard. Mater..

[60] Zhang C., Ai L., Jiang J. (2015). Solvothermal synthesis of MIL–53(Fe) hybrid magnetic composites for photoelectrochemical water oxidation and organic pollutant photodegradation under visible light. J. Mater. Chem. A.

[61] Park H., Oh S., Lee S., Choi S., Oh M. (2019). Cobalt- and nitrogen-codoped porous carbon catalyst made from core–shell type hybrid metal–organic framework (ZIF-L@ZIF-67) and its efficient oxygen reduction reaction (ORR) activity. Appl. Catal. B Environ..

[62] Thakare S.R., Ramteke S.M. (2018). Postmodification of MOF-5 using secondary complex formation using 8-hydroxyquinoline (HOQ) for the development of visible light active photocatalysts. J. Phys. Chem. Solids.

[63] Liu X., Tang B., Long J., Zhang W., Liu X., Mirza Z. (2018). The development of MOFs-based nanomaterials in heterogeneous organocatalysis. Sci. Bull..

[64] Aguilera-Sigalat J., Bradshaw D. (2016). Synthesis and applications of metal-organic framework–quantum dot (QD@MOF) composites. Coord. Chem. Rev..

[65] Müller M., Hermes S., Kähler K., van den Berg M.W.E., Muhler M., Fischer R.A. (2008). Loading of MOF-5 with Cu and ZnO Nanoparticles by Gas-Phase Infiltration with Organometallic Precursors: Properties of Cu/ZnO@MOF-5 as Catalyst for Methanol Synthesis. Chem. Mater..

[66] Kumar A., Chowdhuri A.R., Kumari A., Sahu S.K. (2018). IRMOF-3: A fluorescent nanoscale metal organic frameworks for selective sensing of glucose and Fe (III) ions without any modification. Mater. Sci. Eng. C.

[67] Tanabe K.K., Wang Z., Cohen S.M. (2008). Systematic Functionalization of a Metal−Organic Framework via a Postsynthetic Modification Approach. J. Am. Chem. Soc..

[68] Kim J., McNamara N.D., Her T.H., Hicks J.C. (2013). Carbothermal Reduction of Ti-Modified IRMOF-3: An Adaptable Synthetic Method to Support Catalytic Nanoparticles on Carbon. ACS Appl. Mater. Interfaces.

[69] Saha D., Sen R., Maity T., Koner S. (2013). Anchoring of Palladium onto Surface of Porous Metal–Organic Framework Through Post-Synthesis Modification and Studies on Suzuki and Stille Coupling Reactions Under Heterogeneous Condition. Langmuir.

[70] Bhardwaj N., Bhardwaj S., Mehta J., Kim K.H., Deep A. (2016). Highly sensitive detection of dipicolinic acid with a water-dispersible terbium-metal organic framework. Biosens. Bioelectron..

[71] Sun H., Su H., Ma X., Zhang P., Zhang X., Dai X., Gao J., Chen C., Sun S.-G. (2016). Fe/IRMOF-3 derived porous carbons as non-precious metal electrocatalysts with high activity and stability towards oxygen reduction reaction. Electrochim. Acta.

[72] Lee S.D., Park G.A., Kim D.W., Park D.W. (2014). Catalytic performance of functionalized IRMOF-3 for the synthesis of glycerol carbonate from glycerol and urea. J. Nanosci. Nanotechnol..

[73] Zhou X., Zhang Y., Yang X., Zhao L., Wang G. (2012). Functionalized IRMOF-3 as efficient heterogeneous catalyst for the synthesis of cyclic carbonates. J. Mol. Catal. A Chem..

[74] Chowdhuri A.R., Singh T., Ghosh S.K., Sahu S.K. (2016). Carbon Dots Embedded Magnetic Nanoparticles @Chitosan @Metal Organic Framework as a Nanoprobe for pH Sensitive Targeted Anticancer Drug Delivery. ACS Appl. Mater. Interfaces.

[75] Karra J.R., Walton K.S. (2010). Molecular Simulations and Experimental Studies of CO_2_, CO, and N_2_ Adsorption in Metal−Organic Frameworks. J. Phys. Chem. C.

[76] Ding S., Dong Q., Hu J., Xiao W., Liu X., Liao L., Zhang N. (2016). Enhanced selective adsorption of CO_2_ on nitrogen-doped porous carbon monoliths derived from IRMOF-3. Chem. Commun..

[77] Zahmatkesh M., Ebrahimi S. (2010). Biodegradation of Reactive orange 16 by Phanerochaete chrysosporium fungus: Application in a fluidized bed bioreactor. Iran. J. Environ. Health Sci. Eng..

[78] Albert M., Lessin M.S., Gilchrist B.F. (2003). Methylene blue: Dangerous dye for neonates. J. Pediatr. Surg..

[79] Khan R., Tahir H., Uddin F., Hameed U. (2005). Adsorption of methylene blue from aqueous solution on the surface of wool fiber and cotton fiber. J. Appl. Sci. Environ. Manag..

[80] Phaltane S.A., Vanalakar S.A., Bhat T.S., Patil P.S., Sartale S.D., Kadam L.D. (2017). Photocatalytic degradation of methylene blue by hydrothermally synthesized CZTS nanoparticles. J. Mater. Sci. Mater. Electron..

[81] Yamashita H., Harada M., Misaka J., Takeuchi M., Neppolian B., Anpo M. (2003). Photocatalytic degradation of organic compounds diluted inwater using visible light-responsive metal ion-implanted TiO_2_ catalysts: Fe ion-implanted TiO_2_. Catal. Today.

[82] Pachfule P., Chen Y., Jiang J., Banerjee R. (2012). Fluorinated Metal-Organic Frameworks: Advantageous for Higher H_2_ and CO_2_ Adsorption or Not?. Chem. Eur. J.

[83] Zheng J., Li S., Wang Y., Li L., Su C., Liu H., Zhu F., Jiang R., Ouyang G. (2014). In situ growth of IRMOF-3 combined with ionic liquids to prepare solid-phase microextraction fibers. Anal. Chim. Acta.

[84] Isobe T., Arai Y., Yanagida S., Matsushita S., Nakajima A. (2017). Solvothermal preparation and gas permeability of an IRMOF-3 membrane. Microp. Mesoporous Mater..

[85] Lee Y.-R., Cho S.-M., Ahn W.-S., Lee C.-H., Lee K.-H., Cho W.-S. (2015). Facile synthesis of an IRMOF-3 membrane on porous Al_2_O_3_ substrate via a sonochemical route. Microp. Mesoporous Mater..

[86] Zhong Lin W. (2004). Zinc oxide nanostructures: Growth, properties and applications. J. Phys. Condens. Matter.

[87] Dan-Hardi M., Serre C., Frot T., Rozes L., Maurin G., Sanchez C., Férey G. (2009). A New Photoactive Crystalline Highly Porous Titanium(IV) Dicarboxylate. J. Am. Chem. Soc..

[88] Pham H.Q., Mai T., Pham-Tran N.-N., Kawazoe Y., Mizuseki H., Nguyen-Manh D. (2014). Engineering of Band Gap in Metal–Organic Frameworks by Functionalizing Organic Linker: A Systematic Density Functional Theory Investigation. J. Phys. Chem. C.

[89] Abdelhameed R.M., Carlos L.D., Silva A.M.S., Rocha J. (2015). Engineering lanthanide-optical centres in IRMOF-3 by post-synthetic modification. New J. Chem..

[90] Ma M., Zacher D., Zhang X., Fischer R.A., Metzler-Nolte N. (2011). A Method for the Preparation of Highly Porous, Nanosized Crystals of Isoreticular Metal−Organic Frameworks. Cryst. Growth Des..

[91] Wang X.-L., Fan H.-L., Tian Z., He E.-Y., Li Y., Shangguan J. (2014). Adsorptive removal of sulfur compounds using IRMOF-3 at ambient temperature. Appl. Surf. Sci..

[92] Zhao M., Deng K., He L., Liu Y., Li G., Zhao H., Tang Z. (2014). Core–Shell Palladium Nanoparticle@Metal–Organic Frameworks as Multifunctional Catalysts for Cascade Reactions. J. Am. Chem. Soc..

[93] Yoo Y., Jeong H.-K. (2010). Heteroepitaxial Growth of Isoreticular Metal−Organic Frameworks and Their Hybrid Films. Cryst. Growth Des..

[94] Frisch M.J., Trucks G.W., Schlegel H.B., Scuseria G.E., Robb M.A., Cheeseman J.R., Scalmani G., Barone V., Mennucci B., Petersson G.A. (2009). Gaussian 09.

